# Targeting PPARα in low ambient temperature exposure-induced cardiac dysfunction and remodeling

**DOI:** 10.1186/s40779-021-00347-y

**Published:** 2021-10-19

**Authors:** Xi-Yao Chen, Yang Jiao, Fu-Yang Zhang

**Affiliations:** 1 Department of Geriatrics, Xijing Hospital, Air Force Medical University, Xi’an, 710032 China; 2 Department of Stomatology, The 7th Medical Center of Chinese, PLA General Hospital, Beijing, 100700 China; 3 Department of Cardiology, Xijing Hospital, Air Force Medical University, Xi’an, 710032 China

**Keywords:** Low ambient temperature, Cardiac dysfunction, Remodeling, Peroxisome proliferator-activated receptor-α, Fatty acid metabolism

## Abstract

The present study demonstrates that the down-regulation of peroxisome proliferator-activated receptor-α (PPARα) results in chronic low ambient temperature (LT) exposure-induced cardiac dysfunction and remodeling, emphasizing the therapeutic potential of PPARα activation strategies (e.g. fenofibrate treatment) in LT-associated cardiac injury.

**Supplementary Information:**

The online version contains supplementary material available at 10.1186/s40779-021-00347-y.

## Dear Editor,

Military agents often patrol and carry out combat missions in high-altitude and extremely cold regions. Long-term exposure to low ambient temperature (LT) leads to cardiac contractile dysfunction and pathological structural remodeling [1]. Unfortunately, the underlying mechanisms remain elusive, and effective therapies are urgently needed. Metabolic reprogramming is widely observed in the diseased heart, which allows the myocardial substrate preference to shift from fatty acids (FAs) to glucose utilization [2]. However, the impact of chronic LT exposure on myocardial substrate metabolism has yet to be defined.

The detailed methods and results are available in Additional file 1. Adult C57BL/6J mice were randomly exposed to room temperature (RT, 24–26 °C) or LT (4 °C) for 8 weeks. We found that myocardial metabolic patterns were robustly changed in LT-stressed mice compared with the RT group. The mRNA levels of genes involved in glycolysis (*Hk2*, *Pfkm*, *Pkm*, *Ldha*, and *Pdk4*) were upregulated, whereas the mRNA levels of genes participating in glucose oxidation (*Pdha*, *Pdhb*, *Idh1*, *Ogdh*, and *Suclg2*) and FA metabolism (*Cd36*, *Fabp3*, *Acsl*, *Cpt1b*, *Acaa2*, and *Acadm*) were downregulated in the hearts of mice exposed to LT (Additional file 1: Fig. S1a). The detailed sequences of the primers utilized in the study are available in Additional file 1: Table S1. Peroxisome proliferator-activated receptor-α (PPARα) is a nuclear receptor that transcriptionally regulates FA metabolic gene expression in the heart [3]. Compared with the RT group, the mRNA and protein levels of PPARα were markedly downregulated in the hearts of LT-treated mice (Additional file 1: Fig. S1b). To clarify the role of PPARα in LT-associated cardiac injury, *Ppara*^−/−^ mice and their wild-type (WT) littermates were exposed to RT or LT for 8 weeks. In response to LT, the mRNA levels of genes involved in FA metabolism were much lower in PPARα-deficient hearts than in WT hearts (Additional file 1: Fig. S1c). These results suggest that the downregulation of PPARα contributes to the suppression of FA metabolic gene expression in response to LT. As expected, WT-LT mice exhibited cardiac hypertrophy and lung edema, as indicated by increased heart weight to tibia length ratios (HW/TL) and wet to dry lung weight ratios in comparison to WT-RT mice (Additional file 1: Table S2). Somewhat to our surprise, the cardiac hypertrophy and lung edema were greatly exacerbated in *Ppara*^−/−^-LT mice when compared with WT-LT mice (Additional file 1: Table S2). Echocardiography showed that WT-LT mice suffered from cardiac contractile dysfunction, as evidenced by decreased left ventricular ejection fraction (LVEF) and fraction shortening (FS) values (Additional file 1: Table S2). Compared with WT-LT mice, the cardiac contractile dysfunction was markedly aggravated in *Ppara*^−/−^-LT mice (Additional file 1: Table S2). Molecular analysis showed that the mRNA levels of fetal (*Nppa*, *Nppb*, and *Myh7*) and profibrotic (*Col1a1* and *Col3a1*) genes were much higher in the hearts of LT-treated *Ppara*^−/−^ mice than those in WT hearts (Additional file 1: Fig. S1d). Compared with WT controls, the chronic LT exposure-induced cardiomyocyte hypertrophy and interstitial fibrosis were greatly worsened in *Ppara*^−/−^ mice (Additional file 1: Fig. S1e and f). Together, these data highlight for the first time that the downregulation of PPARα contributes to LT-associated cardiac dysfunction and remodeling.

Next, we investigated whether pharmacological activation of PPARα ameliorates LT-related cardiac injury. C57BL/6J mice were randomized to receive vehicle or fenofibrate [a specific PPARα agonist, 200 mg/(kg day)] when they were exposed to LT [3]. Notably, fenofibrate significantly preserved myocardial FA metabolic gene expression in response to chronic LT exposure (Additional file 1: Fig. S1g). Compared with the vehicle group, fenofibrate ameliorated LT-induced cardiac hypertrophy and lung edema (Additional file 1: Table S3). Fenofibrate markedly eased LT-induced cardiac contractile dysfunction, as evidenced by elevated LVEF and FS values (Additional file 1: Table S3). The upregulation of fetal and profibrotic gene expression induced by LT was greatly attenuated by fenofibrate (Additional file 1: Fig. S1h). Structural analysis showed that fenofibrate mitigated LT-induced cardiomyocyte hypertrophy and interstitial fibrosis (Additional file 1: Fig. S1i). These results demonstrate that pharmacological activation of PPARα might be a promising therapeutic strategy for LT-related cardiomyopathic phenotypes.

LT has been recognized as a neglected health threat for military agents garrisoned in high altitude and high cold regions. Long-term exposure to LT results in cardiac contractile dysfunction and structural remodeling [1]. However, effective therapies are still lacking. FAs are the predominant energy substrates utilized by the heart, and impaired FA metabolism due to the downregulation of PPARα has been widely observed in the failing heart [2]. PPARα-null hearts are protected against ischemia/reperfusion injury and ischemic cardiomyopathy because PPARα deletion suppresses myocardial FA oxidation, reduces the generation of lipotoxic molecules and reactive oxygen species, ameliorates cardiomyocyte apoptosis, and ultimately limits the expansion of the infarcted area [4]. In contrast, in hearts stressed by non-ischemic insults, the downregulation of PPARα results in insufficient energy supply, worsens cardiac dysfunction, and accelerates the development of heart failure [5]. Here, utilizing genetic mouse models, we provide solid evidence demonstrating for the first time that the downregulation of PPARα is responsible for LT-related cardiac dysfunction and remodeling. More importantly, the present work highlights that the activation of PPARα via clinically available drugs (e.g., fenofibrate) might be a novel and promising strategy for the treatment of LT-related cardiac injury.

## Supplementary Information

**Additional file 1: Fig. S1.** Role of PPARα in LT-related cardiac injury. **Table S1.** Primer sequence for RT-PCR. **Table S2.** General biometric and echocardiographic properties of WT and *Ppara*^−/−^ mice upon 8-week room temperature or low ambient exposure. **Table S3.** General biometric and echocardiographic properties of C57BL/6J mice received vehicle or fenofibrate treatment upon 8-wk room temperature or low ambient temperature exposure.

## Data Availability

Detailed methods and supplementary data are available in online Additional file.

## Abbreviations

Acaa2: Acetyl-CoA acetyltransferase-2
Acadm: Medium-chain specific acyl-CoA dehydrogenase
Acsl: Long-chain acyl-CoA synthetase
Col1α1: Collagen, type I, alpha 1
Col3α1: Collagen, type III, alpha 1
Cpt1b: Carnitine palmitoyltransferase 1b
FA: Fatty acid
Fabp3: Fatty acid binding protein-3
Hk2: Hexokinase-2
HW: Heart weight
HF: Heart failure
KO: Knockout
mRNA: Messenger RNA
Myh7: Myosin heavy chain-7
Nppa: Natriuretic peptide precursor a
Nppb: Natriuretic peptide precursor b
PPARα: Peroxisome proliferator activated receptor-α
TL: Tibia length
WT: Wild-type
