# Supplementary material for: Targeting PPARα in low ambient temperature exposure-induced cardiac dysfunction and remodeling
Source: Mil Med Res. 2021 Oct 19;8:53. doi: 10.1186/s40779-021-00347-y (PMC8524846; doi:10.1186/s40779-021-00347-y)
Supplement: Supplementary file 1 — Additional file 1: Fig. S1. Role of PPARα in LT-related cardiac injury. Table S1. Primer sequence for RT-PCR. Table S2. General biometric and echocardiographic properties of WT and Ppara−/− mice upon 8-week room temperature or low ambient exposure. Table S3. General biometric and echocardiographic properties of C57BL/6J mice received vehicle or fenofibrate treatment upon 8-wk room temperature or low ambient temperature exposure. [file 40779_2021_347_MOESM1_ESM.docx]

**Methods and materials**

**Animal group, low ambient temperature exposure and drug treatment**

All the animal procedures in this study were approved by the Animal Care and Use Committee of Air Force Medical University (NO. 2019-0821-7). Global PPARα knockout (*Ppara*^-/-^) mice were commercially obtained from Shanghai Model Organisms (Shanghai, China). 10 to 14-week-old male *Ppara*^-/-^ mice (*n* = 22) and their wild-type (WT) littermates (*n* = 22) were housed at room temperature (RT, 24℃- 26℃) or low ambient temperature (LT, 4℃) within animal facility with free access to food and water for 8 weeks before myocardial function and structure were systemically evaluated [1]. The animal groups were as follows: WT-RT (*n* = 10), *Ppara*^-/-^-RT (*n* = 10), WT-LT (*n* = 12), and *Ppara*^-/-^-LT (*n* = 12). To testify the effect of PPARα agonist on LT-associated cardiac injury, 10 to 12-week-old male C57BL/6J mice (*n* = 44) were housed at RT and LT for 8 weeks and were randomized to receive vehicle or the selective PPARα agonist fenofibrate [200 mg/(kg∙d)] by oral gavage [2]. The groups were as follows: Vehicle-RT (*n* = 10), Fenofibrate-RT (*n* = 10), Vehicle-LT (*n* = 12), and Fenofibrate-LT (*n* = 12). Fenofibrate was commercially obtained (MedChemExpress, Shanghai, China). At the end of the study, systolic and diastolic blood pressures were measured with a semi-automated, non-invasive tail-cuff device. Mice were fully anesthetized with inhaling 1.5% isoflurane and were sacrificed by cutting the carotid artery. Body weights, heart weights, wet and dry lung weights as well as tibia lengths were measured. Heart tissues were rapidly frozen for biochemical analyzes or were fixed within formalin solution for histological analyzes.

**Echocardiography**

Cardiac contractile function was analyzed by M-mode echocardiography with the VEVO 2100 small animal echo system (VisualSonics, Toronto, Canada) as we previously described [3]. In brief, mice were inhaling 1.5% isoflurane and two-dimensional echocardiographic views of the mid-ventricular short axis were collected at the section of the papillary muscle tips below the mitral valve. Subsequent calculation of the left ventricular ejection fraction (LVEF) and fractional shortening (FS) values were performed.

**Real-time quantification PCR**

Total RNA was extracted from frozen heart tissues and RNA reverse-transcription was performed as we previously described [4]. Real-time quantification PCR was performed using a SyBR Green Master Mix (Cowin Biotech, Beijing, China). Primer sequences were listed in **Table S1**.

**Western blotting**

Heart tissues were lysed in RIPA lysis buffer (P0013K, Beyotime, China) containing protease inhibitor cocktail (P1010, Beyotime, China) and phosphatase inhibitor cocktail (#5870, CST, USA). Proteins were separated on 10% SDS-PAGE gels and transferred to PVDF membranes (Millipore, USA). Membranes were blocked in 5% non-fat milk for 1 h at RT, followed by incubation with primary antibodies at 4 ℃ overnight. Protein bands were detected using horseradish peroxidase (HRP)-conjugated secondary antibodies (Cowin Biotech, Beijing, China) and ECL substrate (Millipore, USA). β-actin was used as the loading control. The primary antibodies were as follows: anti-PPARα (1:1000, ab215270, Abcam, USA) and anti-β-actin (1:1000, CW0263, Cowin Biotech, China). After incubation in the secondary antibodies, blots were visualized using enhanced chemiluminescence (Millipore, USA) and scanned by ChemiDocXRS system (Bio-Rad Laboratory, USA).

**Histological analyses**

Cardiac structural analyzes were performed as we previously described [5]. In brief, heart tissues were fixed with formalin solution overnight and embedded in paraffin. 6-μm-thick sections were stained with wheat germ agglutinin (WGA) to measure cardiomyocyte cross-sectional areas, or with masson trichrome to evaluate interstitial fibrosis areas. Staining results were quantified by the ImageJ software (ImageJ, USA).

**Statistical analyses**

Data were presented as mean ± standard error of mean (SEM) and were analyzed by Graphpad Prism 8 software (Graphpad prism, USA). Statistical comparisons between two groups were performed by unpaired student’s *t*-test. For more than two groups, two-way ANOVA followed by the Bonferroni *post-hoc* test was used. A *P* < 0.05 was considered statistically significant.

**
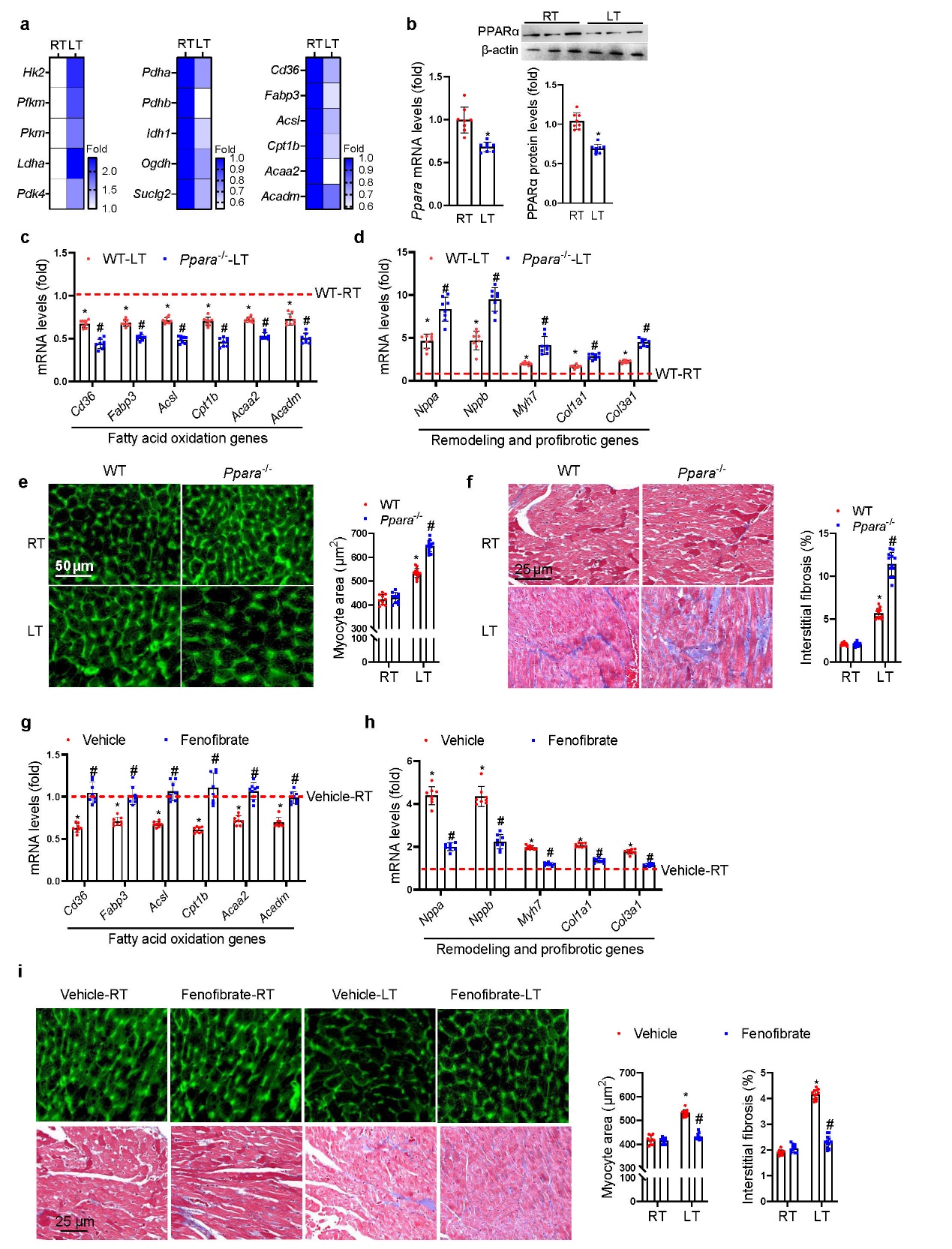
**

**Fig. S1 Role of PPARα in LT-related cardiac injury.** C57BL/6J mice were subjected to room temperature (RT) or low ambient temperature (LT) for 8 weeks. **(a)** mRNA levels of genes involved in glycolysis, glucose oxidation, and fatty acid (FA) metabolism (*n* = 8). **(b)** mRNA and protein expression of cardiac PPARα. WT and *Ppara*^-/-^ mice were subjected to RT or LT for 8 weeks (*n* = 8). **(c)** mRNA levels of FA metabolic genes (*n* = 8). **(d)** mRNA levels of fetal and profibrotic genes. (*n* = 8). **(e)** Myocyte cross-sectional areas (WGA staining, *n* = 10 in WT-RT and *Ppara*^-/-^-RT group, *n* = 12 in WT-LT and *Ppara*^-/-^-LT group). **(f)** Interstitial fibrosis areas (Masson trichrome staining, *n* = 10 in WT-RT and *Ppara*^-/-^-RT group, *n* = 12 in WT-LT and *Ppara*^-/-^-LT group). C57BL/6J mice were subjected to RT or LT for 8 weeks, with or without oral gavage of fenofibrate (*n* = 8). **(g)** mRNA levels of FA metabolic genes (*n* = 8). **(h)** mRNA expression of fetal and profibrotic genes (*n* = 8). **(i)** Myocyte cross-sectional areas (WGA staining) and interstitial fibrosis areas (Masson trichrome staining, *n*=10 in Vehicle-RT and Fenofibrate-RT group, *n* = 12 in Vehicle-LT and Fenofibrate-LT group). Data are shown as the mean ± SEM. **P* < 0.05 *vs*. WT-RT or Vehicle-RT group, ^#^*P* < 0.05 *vs*. WT-LT or Vehicle-LT group. **(a)** and **(b)** were analyzed by unpaired student’s *t*-test. The other data were analyzed by two-way ANOVA followed by the Bonferroni *post-hoc* test.

**Table S1** Primer sequences for RT-PCR

| **Gene** | **Forward (5’ to 3’)** | **Reverse (5’ to 3’)** |
| --- | --- | --- |
| *Cpt1b* | TACACGCATCCCAGGCAAAG | CGAGCCCTCATAGAGCCAAAC |
| *Acsl1* | TTCGCAGTGGCATCGTCAG | TGTGATCATCAGCCGGACTTTC |
| *Acaa2* | ACACCTGGTTCACGAGTTAAG | GTTCTGGATGATCAGGGAGATG |
| *Acadm* | GCTACAAGGTCCTGAGAAGTG | CTCCGTCAACTCGAAGCTAAA |
| *Cd36* | TCAGAACCTATCGAAGGCTTGAATC | AGCTGGCTTGACCAGTATGTTGAC |
| *Fabp3* | TAGCATGACCAAGCCGACCA | ACCAGTTTGCCTCCGTCCAG |
| *Pdha* | TCAATGCACATGTACGCCAAGA | TTATACTTGCAGGCCAGAGCAATTC |
| *Pdhb* | CACATCACTGTAGTTGCCCATTC | ATAGCTTCAATGTCCATTGGTCTG |
| *Idh1* | GAGGCTTCATCTGGGCCTGTAA | CATGGGCAGCCTCTGCTTCTA |
| *Ogdh* | CTGGCCAGGGTATCGTGTATGAG | CATCCGAGGGTCTGTGGTGA |
| *Suclg2* | GTGTAAAGGAGTCCCAAGTCTATC | GTTGACGATCCCACCAAAGA |
| *Hk2* | TCGATGGCTCCGTCTACAAGAA | ACATCACAGTCGGGCACCAG |
| *Pfkm* | GGATATGATACCAGGGTCACTGTTC | AAAGTGCCATCACTGCTTCCA |
| *Pkm* | CGATCTGTGGAGATGCTGAAGG | GGCTGCACGGACATTCTTGA |
| *Ldha* | GACTTGGCCGAGAGCATAAT | GGAAGACATCCTCCTTGATTCC |
| *Ppara* | TGCTGAAGTACGGTGTGTATG | CTTTAGGAACTCTCGGGTGATG |
| *Nppa* | GTGCGGTGTCCAACACAGAT | TCCAATCCTGTCAATCCTACCC |
| *Nppb* | GAGGTCACTCCTATCCTCTGG | GCCATTTCCTCCGACTTTTCTC |
| *Myh7* | ACTGTCAACACTAAGAGGGTCA | TTGGATGATTTGATCTTCCAGGG |
| *Col1a1* | GCTCCTCTTAGGGGCCACT | CCACGTCTCACCATTGGGG |
| *Col3a1* | CTGTAACATGGAAACTGGGGAAA | CCATAGCTGAACTGAAAACCACC |
| *Actin* | GGCTGTATTCCCCTCCATCG | CCAGTTGGTAACAATGCCATGT |

**Table S2** General biometric and echocardiographic properties of WT and *Ppara*^-/-^ mice upon 8-week room temperature or low ambient exposure

| **Biological parameters** | **WT-RT**  **(*n* = 10)** | ***Ppara*^-/-^-RT**  **(*n* = 10)** | **WT-LT**  **(*n* = 16)** | ***Ppara*^-/-^-LT**  **(*n* = 16)** |
| --- | --- | --- | --- | --- |
| Body weight (g) | 29.7 ± 2.6 | 29.3 ± 2.4 | 25.3 ± 1.9^*^ | 25.1 ± 2.6^*^ |
| Systolic BP (mmHg) | 102 ± 11 | 103 ± 10 | 138 ± 15^*^ | 140 ± 14^*^ |
| Diastolic BP (mmHg) | 73 ± 7 | 72 ± 6 | 103 ± 9^*^ | 107 ± 11^*^ |
| HW/TL (mg/mm) | 5.1 ± 0.8 | 5.2 ± 0.7 | 6.3 ± 0.9^*^ | 7.4 ± 1.1^*#^ |
| Lung weight (wet/dry) | 2.1 ± 0.3 | 2.2 ± 0.4 | 4.5 ± 0.7^*^ | 6.7 ± 0.9^*#^ |
| Heart rate (beat/min) | 455 ± 23 | 461 ± 19 | 472 ± 26 | 476 ± 29 |
| LVEDD (mm) | 2.5 ± 0.1 | 2.5 ± 0.1 | 2.8 ± 0.1^*^ | 3.5 ± 0.2^*#^ |
| LVESD (mm) | 1.5 ± 0.1 | 1.6 ± 0.1 | 1.9 ± 0.1^*^ | 2.6 ± 0.1^*#^ |
| FS (%) | 39.6 ± 2.1 | 36.9 ± 2.9 | 32.2 ± 4.1^*^ | 26.3 ± 3.9^*#^ |
| LVEF (%) | 73.6 ± 3.5 | 72.5 ± 4.2 | 60.1 ± 5.6^*^ | 51.4 ± 6.1^*#^ |

*BP* blood pressure; *LVEF* left ventricular ejection fraction. *FS* fractional shortening; *HW* heart weight; *TL* tibia length; *LVEDD* left ventricular end-diastolic diameter; *LVESD* left ventricular end-systolic diameter; *WT* wild-type; *RT* room temperature; *LT* low ambient temperature. ^*^*P* < 0.05 *vs*. WT-RT group, ^#^*P* < 0.05 *vs*. WT-LT group. Data are shown as mean ± SD and analyzed by two-way ANOVA followed by the Bonferroni *post-hoc* test.

**Table S3** General biometric and echocardiographic properties of C57BL/6J mice received vehicle or fenofibrate treatment upon 8-week room temperature or low ambient temperature exposure

| **Biological parameters** | **Vehicle-RT**  **(*n* = 10)** | **Fenofibrate -RT**  **(*n* = 10)** | **Vehicle-LT**  **(*n* = 16)** | **Fenofibrate -LT**  **(*n* = 16)** |
| --- | --- | --- | --- | --- |
| Body weight (g) | 28.9 ± 1.9 | 29.1 ± 1.8 | 26.3 ± 2.1^*^ | 25.9 ± 1.8^*^ |
| Systolic BP (mmHg) | 99 ± 7 | 101 ± 8 | 128 ± 13^*^ | 131 ± 15^*^ |
| Diastolic BP (mmHg) | 71 ± 5 | 72 ± 6 | 92 ± 8^*^ | 97 ± 10^*^ |
| HW/TL (mg/mm) | 5.2 ± 0.7 | 5.2 ± 0.6 | 6.4 ± 0.8^*^ | 5.4 ± 0.7^*#^ |
| Lung weight (wet/dry) | 2.0 ± 0.5 | 2.1 ± 0.5 | 4.7 ± 0.8^*^ | 3.2 ± 0.6^*#^ |
| Heart rate (beat/min) | 448 ± 19 | 452 ± 21 | 466 ± 18 | 463 ± 21 |
| LVEDD (mm) | 2.4 ± 0.1 | 2.4 ± 0.1 | 2.9 ± 0.1^*^ | 2.5 ± 0.1^#^ |
| LVESD (mm) | 1.5 ± 0.1 | 1.5 ± 0.1 | 2.0 ± 0.1^*^ | 1.6 ± 0.1^*#^ |
| FS (%) | 37.5 ± 1.9 | 37.4 ± 2.1 | 31.3 ± 3.2^*^ | 36.3 ± 2.8^*#^ |
| LVEF (%) | 74.1 ± 4.2 | 73.7 ± 3.8 | 59.4 ± 4.6^*^ | 67.4 ± 4.1^*#^ |

*BP* blood pressure; *LVEF* left ventricular ejection fraction. *FS* fractional shortening; *HW* heart weight; *TL* tibia length; *LVEDD* left ventricular end-diastolic diameter; *LVESD* left ventricular end-systolic diameter; *LT* low ambient temperature; *RT* room temperature. ^*^*P* < 0.05 *vs*. Vehicle-RT group, ^#^*P* < 0.05 *vs*. Vehicle-LT group. Data are shown as mean ± SD and analyzed by two-way ANOVA followed by the Bonferroni *post-hoc* test.

**References:**

1. Yin Z, Ding G, Chen X, Qin X, Xu H, Zeng B, et al. Beclin1 haploinsufficiency rescues low ambient temperature-induced cardiac remodeling and contractile dysfunction through inhibition of ferroptosis and mitochondrial injury. Metabolism. 2020;113:154397.

2. Colin S, Briand O, Touche V, Wouters K, Baron M, Pattou F, et al. Activation of intestinal peroxisome proliferator-activated receptor-α increases high-density lipoprotein production. Eur Heart J. 2013;34(32):2566-74.

3. Zhang F, Wang K, Zhang S, Li J, Fan R, Chen X, et al. Accelerated FASTK mRNA degradation induced by oxidative stress is responsible for the destroyed myocardial mitochondrial gene expression and respiratory function in alcoholic cardiomyopathy. Redox Biol. 2021;38:101778.

4. Li Y, Xiong Z, Yan W, Gao E, Cheng H, Wu G, et al. Branched chain amino acids exacerbate myocardial ischemia/reperfusion vulnerability via enhancing GCN2/ATF6/PPAR-α pathway-dependent fatty acid oxidation. Theranostics. 2020;10(12):5623-40.

5. Zhang F, Xia Y, Yan W, Zhang H, Zhou F, Zhao S, et al. Sphingosine 1-phosphate signaling contributes to cardiac inflammation, dysfunction, and remodeling following myocardial infarction. Am J Physiol Heart Circ Physiol. 2016;310(2):H250-61.
